# The Role of Cognitive Behavioral Therapy in Opioid Use Reduction in Pediatric Sickle Cell Disease: Protocol for a Systematic Review

**DOI:** 10.2196/13211

**Published:** 2019-07-17

**Authors:** Ashaunta T Anderson, Nhu Tran, Kathryn Smith, Lorraine I Kelley-Quon

**Affiliations:** 1 Division of General Pediatrics Children’s Hospital Los Angeles Los Angeles, CA United States; 2 Department of Pediatrics University of Southern California Keck School of Medicine Los Angeles, CA United States; 3 Department of Surgery Children's Hospital Los Angeles Los Angeles, CA United States; 4 Department of Surgery University of Southern California Keck School of Medicine Los Angeles, CA United States; 5 Department of Preventive Medicine University of Southern California Keck School of Medicine Los Angeles, CA United States

**Keywords:** sickle cell disease, sickle cell anemia, pain, opioids, cognitive behavioral therapy, CBT, children, adolescents, youth

## Abstract

**Background:**

Sickle cell disease (SCD) is a genetic disorder of red blood cells that results in acute and chronic health problems, including painful syndromes. Opioid analgesia is the mainstay of moderate to severe pain management in SCD, although adjunctive psychosocial approaches such as cognitive behavioral therapy (CBT) are increasingly incorporated. CBT has been used in populations of various ages to address a wide range of issues, such as mood disorders and chronic pain. It is unclear if effective CBT reduces the use of opioids to manage pain in pediatric SCD.

**Objective:**

The aim of this study is to evaluate the association between CBT and decreased opioid use in children with SCD.

**Methods:**

In this systematic review protocol, we describe our approach to applying predetermined eligibility criteria to searches of PubMed (including Medline), Embase, Cochrane, Web of Science, and PsycINFO databases, as well as Google Scholar and grey literature. In particular, we will use keywords to search for English-language studies of individuals with SCD aged 21 years old and younger published before November 2018. Keywords will allow us to assess for the primary outcome—total use of opioid medications—and the secondary outcomes—pain intensity and emotional functioning—during pain management using a combined opioid and CBT approach, opioids alone, or CBT alone. The review team will use standardized abstraction forms to review articles at the title, abstract, and full-text levels. Finally, reviewers will assess the risk for bias, quality of evidence, and adequacy of data for quantitative versus qualitative synthesis. If meta-analysis is deemed inappropriate, a narrative review will be conducted.

**Results:**

We will report a summary of findings across studies that meet eligibility criteria to compare the extent to which adjunctive CBT is associated with decreased opioid use among children with SCD.

**Conclusions:**

This systematic review will present the current state of the evidence on CBT and opioid use in pediatric SCD, which may inform clinical practice and health policy to support optimized pain management.

**International Registered Report Identifier (IRRID):**

PRR1-10.2196/13211

## Introduction

Sickle cell disease (SCD) is an inherited form of severe anemia that affects nearly 100,000 individuals in the United States [1], including 1 in 400 African Americans and 1 in 19,000 Latinos [2]. The clinical hallmark of SCD is the acute painful episode, although the severity and chronicity of pain, end-organ complications, and requirement for frequent contacts with the health care system place children at risk for several psychosocial problems. In particular, children with SCD are at risk for psychological complications, including depression, anxiety, reduced quality of life, and neurocognitive impairment [3,4]. However, current clinical guidelines rely on sparse literature to address the psychological well-being of children with SCD.

Frequent acute painful episodes and attendant restrictions on daily activities of life require the combination of judicious opioid administration along with socioemotional supports [5]. Such painful events may be acute or chronic, treated on an inpatient or outpatient basis, or managed with opioid or nonopioid medications [6]. However, poorly treated acute pain may evolve into psychological problems, reduced quality of life, chronic opioid use, frequent health care usage, and chronic pain syndromes [7,8]. Studies have shown that up to 30% of adolescents with SCD will ultimately develop chronic pain [9]. The most effective pain management regimens are those that address biological and psychosocial antecedents and consequences of disease manifestations [5,7].

Opioids are addictive, particularly in the adolescent population, which encompasses many children with SCD [5,10]. Although concerns surrounding the opioid epidemic have risen, adjunctive psychosocial interventions, such as cognitive behavioral therapy (CBT), are increasingly considered alongside traditional pharmacologic treatments [11,12]. CBT is a commonly employed form of psychotherapy that helps individuals manage negative thoughts to overcome a wide range of problems, including stress and depression [13]. As this evidence base suggests, children with SCD are at an increased risk for depression and poor coping strategies [14-17], and CBT is a promising adjunct to manage disease-related pain [4,18,19]. Despite this encouraging nascent evidence base, the extent to which CBT decreases the use of opioid analgesia is uncertain. If CBT successfully manages pain related to SCD with decreased reliance on opioids, more financial and clinical support for this psychotherapeutic approach would be warranted.

This systematic review will determine if CBT decreases opioid use in children with SCD. Two reviews have described psychosocial interventions for SCD [19,20], but these included a range of approaches for adults and children with very few targeting the outcome of pain medications [19]. Intervention studies that focus on CBT including children with SCD [18,21,22] and CBT as an adjunct therapy in youth with chronic pain syndromes [23] are foundational to this ongoing work. Our work updates the prior reviews with the presentation of data pertinent to CBT and opioid use for painful episodes in pediatric SCD. A systematic review of the literature will guide clinical and health policy decisions pivotal to effective pain management for children with SCD.

The objective of this study is to evaluate the association between CBT and decreased opioid use in children with SCD.

## Methods

### Eligibility Criteria

The population will include children and youth aged 21 years and younger with SCD, the intervention will be CBT and opioid use, the comparator will be opioid use alone, and the outcome will be decreased pain.

### Information Sources

We will search PubMed (including Medline), Embase, Cochrane, Web of Science, and PsycINFO for English-language articles published before November 2018. In addition, we will search the grey literature and Google Scholar. We will also review the reference list of each chosen article for additional relevant articles. The language will be restricted to English, but there will be no geographical restrictions.

### Search Strategy

The search strategy will include the study population using keywords derived from the team’s expertise. Search terms will include a combination of the following: sickle cell disease, cognitive behavioral therapy, cognitive coping strategies, pain coping skills training, opioids and pain, as well as related terms. We will consult with a psychologist to expand the list of search terms for CBT. We will also work with a librarian from the Keck School of Medicine of the University of Southern California’s Norris Medical Library to ensure the integrity and thoroughness of our literature search.

### Study Records

#### Data Management

After the initial search, all articles will be imported into Covidence reference manager software, and all duplicates will be removed.

#### Article Selection Process

The review team will independently screen the titles and abstracts for relevance to our topic. The team will then review full-text articles for inclusion or exclusion based on our research question. The reference lists of all included articles, systematic reviews, and meta-analyses that look at CBT, opioid use in children, and SCD will be manually reviewed to identify any additional articles, which will then be retrieved and reviewed. We plan to exclude studies that have used other treatment modalities (eg, physical therapy or integrated treatments) in conjunction with CBT or opioids. Articles selected for inclusion will not be limited to randomized controlled trials to increase the number of relevant reports for review.

#### Data Collection Process

Using a standardized data abstraction form, reviewers will independently abstract relevant data related to our research question from all articles that meet the inclusion criteria. The form will include the following data elements: author, publication year, sample size (N), age, study setting, study design, pain management intervention, outcome measures, and results, specifically opioid use, pain intensity, and emotional functioning.

### Data Items

The population to be evaluated includes children aged 21 years and younger with a diagnosis of SCD. Children with SCD who undergo CBT will be compared to children who undergo routine care and pain management. The interventions of interest include CBT alone versus opioids alone versus a combination of CBT and opioid use to manage pain. Funding sources, particularly drug-sponsored trials, will be reported.

### Outcomes and Prioritization

The primary outcome of interest will be the total amount of opioids used during inpatient hospitalization or outpatient care. Of note, there can be significant variation in how quantities of opioids are measured across various studies. When actual dosing is available, a standard measurement of total morphine equivalents will be calculated and compared between studies [24].

The secondary outcomes of interest will expand our review to encompass two patient-centered outcomes meaningful to children with SCD: pain intensity and emotional functioning [25]. First, we will capture reports of pain intensity consistent with the Pediatric Initiative on Methods, Measurement, and Pain Assessment in Clinical Trials (PedIMMPACT) recommendations for core outcome domains and measures for pediatric chronic or recurrent pain. Specifically, we will evaluate papers that include pain intensity assessments using the following measures: (1) The Poker Chip Tool for children aged 3 to 4 years [26], (2) the Faces Pain Scale-revised for children aged 4 to 12 years [27], and (3) the Visual Analog Scale for children aged 8 years and older [28]. Comparisons will be performed between subgroups of children with SCD who underwent CBT compared to children who did not receive CBT. These measures have been previously validated in pediatric sickle cell populations [29]. PedIMMPACT also underscores the need to assess emotional functioning in children with chronic pain; therefore, our review will also evaluate studies of emotional functioning in children with SCD as another secondary outcome, comparing levels of emotional functioning based on receipt of CBT. Studies that include the following emotional functioning assessments will be included: (1) Children’s Depression Inventory for children aged 7 to 17 years [30] or (2) the Revised Child Anxiety and Depression Scale (RCADS) [31]. Of note, previous literature highlights an association between depressive symptoms and opioid use in youth [32]. Therefore, assessment of emotional functioning in children with SCD may be particularly relevant to our primary outcome of opioid use.

### Risk of Bias in Individual Studies

Two authors (NT and AA) will independently assess the risk of bias in each of the studies. The assessment of the risk for bias will follow the principles outlined in the *Cochrane Handbook for Systematic Reviews of Interventions* [33]. We will verify that studies minimize bias and address biases that were present. The bias assessment will provide the strengths and weaknesses of the study. Each study will be assessed for:

1. Adequate sequence generation to examine if investigators used a random component in their study design.
2. Allocation concealment to assess whether the study team member applying randomization was blinded to the treatment participants will receive.
3. Blinding (subjective outcomes) to ensure that the participants did not unknowingly affect results because of the knowledge of their treatment group.
4. Blinding (mortality) to safeguard that outcomes were not biased by the knowledge of participant deaths.
5. Incomplete outcome data (short-term and long-term outcomes) to evaluate whether the authors accounted for the missing time points and how it could have affected results.
6. Selective reporting to verify if investigators reported all results, not just the results that were significant.

### Confidence in Cumulative Evidence and Data Synthesis

#### Confidence in Cumulative Evidence

Two authors (NT and AA) will independently appraise the quality of evidence for each of the studies. We will use the GRADE (Grading of Recommendations, Assessment, Development and Evaluation) criteria to rate the quality of the studies [34]. High-quality evidence will demonstrate a very large effect, evidence of a dose response, precision, consistency, directness, and lack of publication bias. The quality will be lower if there is:

1. serious (−1) or very serious (−2) risk of bias;
2. serious (−1) or very serious (−2) inconsistencies;
3. serious (−1) or very serious (−2) indirectness;
4. serious (−1) or very serious (−2) imprecision; and
5. likely (−1) or very likely (−2) publication bias.

#### Data Synthesis Plan

If it is appropriate to quantitatively assess the data, we will summarize the difference in means of opioid use among the studies using CBT in children with SCD. We will only compare studies that meet our eligibility criteria. We will combine results of studies with homogeneous study designs for a meta-analysis. We also plan to assess studies for heterogeneity by making pairwise comparisons of studies that have used CBT with the outcomes of pain score or decreased opioid use. Statistical heterogeneity will be identified or quantified by using the inconsistency equation: I^2^=(Q−df/Q) × 100% test, where *Q* is the chi-square statistic and *df* is its degrees of freedom. The results of the I^2^ findings will be graded as follows: (1) 0% to 40% may not be important, (2) 30% to 60% has potential for moderate heterogeneity, (3) 50% to 90% has potential for substantial heterogeneity, and (4) 75% to 100% has sizeable heterogeneity. We will also consider the magnitude of effects and the strength of the associations (*P* ≤.10). If the potential for heterogeneity exists, we will run a random-effects meta-analysis that incorporates the heterogeneity. We will also take precautions in interpreting the results of the meta-analysis. If there is substantial evidence of heterogeneity, we will use a narrative approach for the review. If quantitative data analysis is not appropriate, we will use a narrative synthesis to describe: (1) eligibility criteria, (2) whether CBT reduces opioid use in children with SCD versus only opioid use, (3) whether CBT is associated with pain intensity and emotional functioning in children with SCD, and (4) a summary of findings across the studies.

## Results

We plan to report a summary of the findings across studies that meet eligibility criteria to present the extent to which adjunctive CBT is associated with decreased opioid use among children with SCD. We expect to complete data analysis by December 2019 and publish results in the following calendar year.

## Discussion

This systematic review will synthesize and report the current state of evidence on CBT and opioid use in pediatric SCD, which may inform clinical practice and health policy to support improved management.

## Abbreviations

CBT: cognitive behavioral therapy
GRADE: Grading of Recommendations, Assessment, Development and Evaluation
PedIMMPACT: Pediatric Initiative on Methods, Measurement, and Pain Assessment in Clinical Trials
RCADS: Revised Child Anxiety and Depression Scale
SCD: sickle cell disease
